# Microarray profiling of long non-coding RNA (lncRNA) associated with hypertrophic cardiomyopathy

**DOI:** 10.1186/s12872-015-0056-7

**Published:** 2015-07-04

**Authors:** Wei Yang, Yuan Li, Fawei He, Haixiang Wu

**Affiliations:** Department of Ultrasonics, The Second Hospital of Sichuan, No. 55, People’s South Road, Wuhou District, 610041 Chengdu, Sichuan China

**Keywords:** Hypertrophic cardiomyopathy, LncRNA, Gene ontology, KEGG pathway

## Abstract

**Background:**

Hypertrophic cardiomyopathy (HCM) is an inherited disorder with around 1400 known mutations; however the molecular pathways leading from genotype to phenotype are not fully understood. LncRNAs, which account for approximately 98 % of human genome, are becoming increasingly interesting with regard to various diseases. However, changes in the expression of regulatory lncRNAs in HCM have not yet been reported.

To identify myocardial lncRNAs involved in HCM and characterize their roles in HCM pathogenesis.

**Methods:**

Myocardial tissues were obtained from 7 HCM patients and 5 healthy individuals, and lncRNA and mRNA expression profiles were analyzed using the Arraystar human lncRNA microarray. Real-time PCR was conducted to validate the expression pattern of lncRNA and mRNA. Gene ontology (GO) enrichment and KEGG analysis of mRNAs was conducted to identify the related biological modules and pathologic pathways.

**Results:**

Approximately 1426 lncRNAs (965 up-regulated and 461 down-regulated) and 1715 mRNAs (896 up-regulated and 819 down-regulated) were aberrantly expressed in HCM patients with fold change > 2.0. GO analysis indicated that these lncRNAs–coexpressed mRNAs were targeted to translational process. Pathway analysis indicated that lncRNAs–coexpressed mRNAs were mostly enriched in ribosome and oxidative phosphorylation.

**Conclusion:**

LncRNAs are involved in the pathogenesis of HCM through the modulation of multiple pathogenetic pathways.

**Electronic supplementary material:**

The online version of this article (doi:10.1186/s12872-015-0056-7) contains supplementary material, which is available to authorized users.

## Background

Hypertrophic cardiomyopathy (HCM) represents the most common inherited cardiac disease with an estimated prevalence of 0.2 % in the general population [1, 2]. Classically, HCM is morphologically characterized by varying degrees of myocardial hypertrophy, myocyte disarray and interstitial fibrosis [3, 4]. It is also a leading cause of sudden cardiac death in young people, including athletes [5]. Approximately 25 % of individuals with HCM demonstrate left ventricular outflow tract (LVOT) obstruction [6]. It has been accepted that HCM is caused predominately by genetic variants. To date, around 1400 mutations have been identified as being responsible for HCM pathology and more than 60 % of genetic variants occurred in 9 sarcomeric genes, including *MYH7*, *MYL2* and *MYBPC3* [7–10]. However, the molecular mechanisms that underlie the pathogenesis of HCM remain largely unknown.

Long non-coding RNAs (lncRNAs) are defined as the transcripts of more than 200 nucleotides that structurally resemble mRNAs but do not encode proteins [11]. The Encyclopedia of DNA Elements (ENCODE) project has reported that >90 % of the genome can be transcribed and the non-coding transcripts account for approximately 98 % [12]. Normally, lncRNAs are involved in a variety of biological processes, such as cell-cycle control, differentiation, apoptosis, chromatin remodeling as well as epigenetic regulation [13, 14]. Dysregulation of lncRNAs has been reported in numerous human diseases, including cancer [15] and neurological diseases [16]. However, dysregulation of lncRNAs in patients with HCM has never been reported. In the present study, we hypothesized that lncRNAs were dysregulated in hypertrophied myocardial tissues.

In order to reveal the potential roles of lncRNAs in the pathogenesis of HCM, we performed microarray analysis to identify dysregulated lncRNAs and mRNAs in HCM patients, compared to control subjects.

## Methods

### Ethical statement

All subjects provided written informed consent prior to participation and all procedures in the present study were approved by the Ethics Committee of the second people’s hospital of Sichuan.

### Tissues from patients with HCM and healthy individuals

Hypertrophied myocardial tissues used in this study were obtained from 7 HCM patients (4 males and 3 females) with severe symptoms (New York Heart Association functional classes III or IV) who underwent septal myectomy at outpatient department of Chengdu first people’s hospital (from May 2013 to December 2013). All patients were diagnosed by two experienced clinicians, based on two-dimensional echocardiography demonstrating an unexplained left ventricular hypertrophy (diastolic interventricular septal thickness ≥ 15 mm and the ratio of septal to posterior wall thickness ≥1.3) in the absence of another cardiac or systemic disease capable of producing a similar magnitude of hypertrophy. Besides, 5 normal myocardial tissues, excised from 5 healthy individuals (3 males and 2 females) at autopsy who voluntarily donated their body for research to the Center of Forensic Medicine in West China, were used as a control group. It is worth noting that we didn’t perform genetic scanning of 1400 known candidate genes in patients.

The following data was collected for all subjects: gender, age at diagnosis or death, heart rate, family history of hypertrophic cardiomyopathy, previous angina pectoris, systolic blood pressure (SBP) and diastolic blood pressure (DBP) (Table 1). Diseases that would affect cardiac structures and functions were absent in any individual. These diseases included, but not limited to, primary hypertension with duration of more than 10 years, secondary hypertension, ischemic heart disease, congenital heart disease, rheumatic heart disease, aortic stenosis and other similar diseases.

**Table 1:** Clinical characteristics of HCM patients (*n* = 7) and controls (*n* = 5)

Indices	HCM patients (*N* =7)							Controls (*N* = 5)					*P*-value
	1	2	3	4	5	6	7	1	2	3	4	5	
Gender	F	M	M	F	F	M	M	F	F	M	M	M	0.92
Age (years)	51	39	49	60	42	54	43	48	54	31	41	40	0.29
Family history of hypertrophic cardiomyopathy (Y or N)	Y	N	N	N	Y	Y	Y	N	N	N	N	N	0.04
Heart rate (bpm)	80	98	74	73	70	62	67	74	84	59	61	60	0.29
Previous angina pectoris	N	N	Y	Y	Y	N	N	N	N	N	Y	N	0.41
SBP (mmHg)	114	122	112	117	121	123	115	127	110	126	129	131	0.11
DBP (mmHg)	82	62	65	70	75	74	71	75	80	71	82	91	0.09
NYHA Classes	3	4	3	4	3	3	3	N.A.	N.A.	N.A.	N.A.	N.A.	

*F* female, *M* male, *Y* yes, *N* no, *SBP* systolic blood pressure, *DBP* diastolic blood pressure, *NYHA* New York Heart Association, *N. A.* not available

### RNA extraction

Myocardial specimens were quickly excised and snap-frozen in liquid nitrogen. Tissues were homogenized in TRIZOL reagent (Invitrogen, USA) using a Qiagen Tissuelyser. Total RNA was extracted in accordance with the manufacturer’s protocol and then quantified using a NanoDrop ND-1000 spectrophotometer (Thermo Fisher Scientific, Waltham, MA). RNA integrity of each sample was assessed by denaturing agarose gel electrophoresis.

### RNA labeling and array hybridization

The expression of lncRNAs and mRNA was determined using Arraystar Human LncRNA Microarray v2.0 (CapitalBio, China). Sample labeling and array hybridization were performed according to the Agilent One-Color Microarray-Based Gene Expression Analysis protocol (Agilent Technology, USA) with minor modifications for all 12 samples. First, rRNA was removed from total RNA using mRNA-ONLY™ Eukaryotic mRNA Isolation Kit (Epicentre Biotechnologies, USA). Then, each sample was amplified and transcribed into fluorescent cRNA along the entire length of the transcripts without 3′ bias using the random priming method. The labeled cRNAs were purified using an RNeasy Mini Kit (Qiagen, Germany) and then hybridized with the specific probes on the Human LncRNA Array v2.0. Positive probes for housekeeping genes and negative probes are printed onto the array for quality control. The hybridized arrays were washed, fixed, and scanned with an Agilent DNA Microarray Scanner G2505C.

### Microarray data analysis

Agilent Feature Extraction software (version 11.0.1.1) was used to analyze acquired array images. Quantile normalization and subsequent data processing were performed using the GeneSpring GX software package (version 11.5.1, Agilent Technologies). Differentially expressed lncRNAs and protein-coding mRNAs between patient group and control group were identified through Volcano Plot filtering (fold change > 2 and *P* < 0.05).

### Real-time PCR

Single-strand cDNA was synthesized by AMV Reverse Transcriptase Kit (Promega, USA) in accordance with the manufacturer’s instructions. Real-time PCR was performed using SsoFast EvaGreen Supermix (Bio-Rad, USA) on a CFX96 Real-Time PCR Detection System (Bio-Rad, USA). The PCR conditions included an initial step at 95 °C for 30 s, followed by 40 cycles of amplification and quantification (95 °C for 5 s, 60 °C for 5 s). Each DNA sample was performed in triplicates in a final volume of 25 μl containing 1 μl of cDNA and 400 nM of forward and reverse gene-specific primers. Relative gene expression levels were quantified based on the cycle threshold (Ct) values and ACTIN was used as an internal control. For quantitative results, expression of each gene was represented as a fold change using the following mathematical model: Fold change = (E_target_)^ΔCttarget (Control - Sample)^/(E_ref_)^ΔCtref (Control - Sample)^. E_target_ and E_ref_ are the PCR efficiency of target gene transcript and reference gene transcript, respectively; ΔCt_target_ is the Ct deviation of control – sample of the target gene transcript; ΔCt_ref_ is the Ct deviation of control – sample of the reference gene transcript. All primer pairs are available upon request.

### Functional group analysis

Gene Ontology (GO) analysis was performed to explore the functions of differentially expressed coding genes identified in this study by using the Database for Annotation, Visualization and Integrated Discovery (DAVID; http://david.abcc.ncifcrf.gov/) [17, 18]. Pathway analysis was used to place differentially expressed coding genes according to Kyoto Encyclopedia of Genes and Genomes (KEGG), Biocarta and Reactome (http://www.genome.jp/kegg/). Generally, Fisher’s exact test and v2 test were used to classify the GO category and select the significant pathway. Besides, the threshold of significance was defined by the P-value and false discovery rate (FDR).

### Statistical analysis

The statistical significance of microarray data was analyzed in terms of fold change using the Student’s *t*-test and FDR was calculated to correct the *P*-value. FC ≥ 2 and *P* < 0.05 were used to screen the differentially expressed lncRNAs and coding mRNAs. For other statistical analysis, GraphPad Prism 5 software and Microsoft Office software were applied. Stusent’s *t*-test was applied for comparison of two groups and differences with *P* < 0.05 were considered statistically significant.

## Results

### Analysis of differentially expressed lncRNAs

**Fig. 1:**
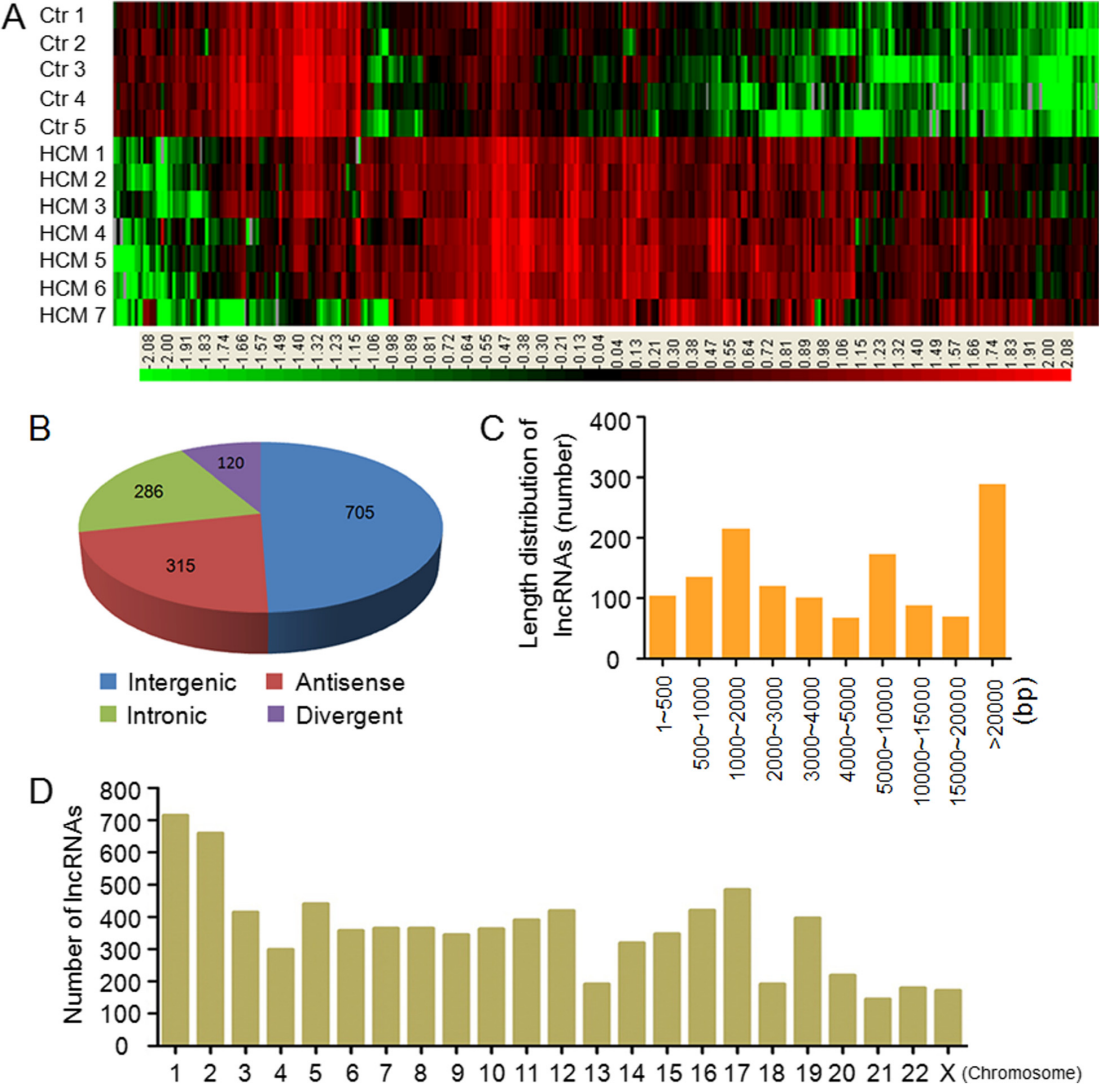
Overview of lncRNAs microarray analysis. **a** The hierarchical clustering of partial differentially expressed lncRNAs between 7 HCM patients and 5 controls. ‘Red’ indicates high relative expression, and ‘green’ indicates low relative expression. Ctr: control. **b** Class distribution of dysregulated lncRNAs, including 705 intergenic, 315 antisense, 286 intronic and 120 divergent. **c** The length distribution of lncRNAs on Chromosome (DNA). The lncRNAs genes were mainly between 200 and 5000 bp in length. **d** Chromosomal distribution of dysregulated lncRNAs

In total, 8435 lncRNAs were detected by Arraystar Human LncRNA Microarray v2.0 (Additional file 1). From the lncRNA expression profiles, differentially expressed lncRNAs were discriminated between patients with HCM and controls. Hierarchical Clustering was performed to group lncRNAs based on their expression levels among samples (Fig. 1a). We set a threshold as fold-change >2.0, *P*-value < 0.05 and FDR < 0.05, and found that a total of 1426 lncRNAs were differentially expressed, consisting of 965 up-regulated lncRNAs and 461 down-regulated lncRNAs. The top 10 up-regulated and 10 down-regulated lncRNAs between two groups were listed in Table 2.

**Table 2:** Top 10 up-regulated and 10 down-regulated lncRNAs between HCM patients and controls

lncRNA ID	FC (abs)	Regulation	FDR	Chrosome	Strand	Start^1^	End^1^	Class	Database
ENST00000472913.1	8.29	up	5.11E–19	3	+	155203591	155204910	Intronic	ENSEMBL
ENST00000588634.1	10.05	up	1.01E–15	19	+	11314303	11326844	Antisense	ENSEMBL
TCONS_00006679	12.76	up	1.99E–10	3	-	148164072	148173245	Intergenic	Human LincRNA Catalog
ENST00000440196.2	12.93	up	3.48E–21	1	-	529832	530597	Intergenic	ENSEMBL
TCONS_00024565	14.61	up	1.60E–18	16	-	3995946	4002465	Intergenic	Human LincRNA Catalog
ENST00000445814.1	14.90	up	1.28E–01	X	-	73047635	73051045	Intergenic	ENSEMBL
TCONS_00017343	15.20	up	8.42E–02	X	-	73040858	73061243	Intergenic	Human LincRNA Catalog
TCONS_00017432	27.97	up	6.45E–02	X	+	73045949	73047819	Intergenic	Human LincRNA Catalog
XR_110349.1	0.13	down	9.57E–15	12	-	49626572	49658539	Divergent	RefSeq
ENST00000450016.1	0.14	down	1.92E–07	7	+	44888657	44889164	Divergent	ENSEMBL
ENST00000443565.1	0.15	down	2.14E–04	1	+	81106968	81112473	Intergenic	ENSEMBL
HIT000075931	0.15	down	6.17E–22	5	-	172083325	172083570	Intronic	H-InvDB
TCONS_00022136	0.16	down	8.21E–09	13	+	20530916	20532202	Divergent	Human LincRNA Catalog
ENST00000376482.3	0.17	down	4.56E–16	7	+	99728586	99738062	Intergenic	ENSEMBL
uc.324-	0.19	down	5.29E–01	11	-	30557520	30557745	Antisense	UCR
TCONS_00014990	0.19	down	2.04E–16	8	-	41998257	41998755	Intergenic	Human LincRNA Catalog
TCONS_00014155	0.20	down	6.11E–14	7	+	65959182	65960476	Intergenic	Human LincRNA Catalog
ENST00000563833.1	0.20	down	3.97E–09	4	-	103421997	103422476	Divergent	ENSEMBL

*FC* fold change, *FDR* false discover rate

^1^Chromosomal positions based on GRCh38

We summarized the classification and length distribution of dysregulated lncRNAs. Among the dysregulated lncRNAs, there were 705 intergenic, 315 antisense, 286 intronic and 120 divergent (Fig. 1b). These differentially expressed lncRNAs showed different length on chromosome (DNA) ranging from 86 bp to 506 kb and 753 lncRNAs (52.8 %) were between 200 bp and 5000 bp in length (Fig. 1c). Moreover, these differentially expressed lncRNAs were distributed across nearly all of the human chromosomes (Fig. 1d).

### Analysis of differentially expressed mRNAs

**Fig. 2:**
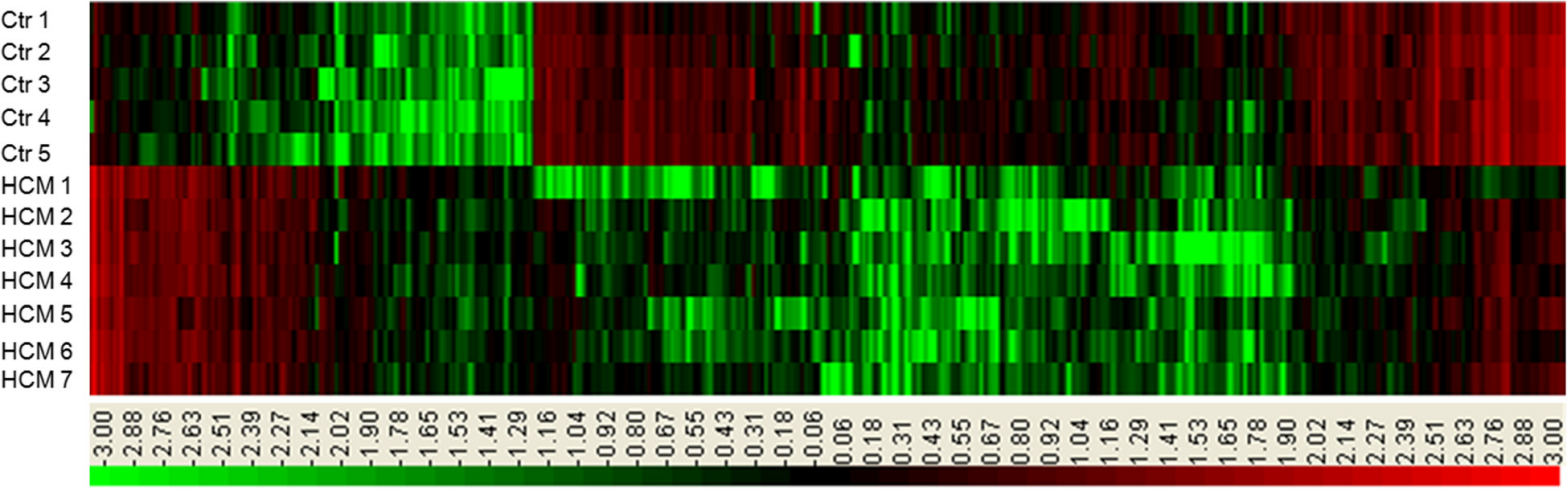
The hierarchical clustering of partial differentially expressed mRNAs. ‘Red’ indicates high relative expression, and ‘green’ indicates low relative expression. Ctr: control

From the analysis, we totally detected 8404 mRNAs, of which 1715 showed significantly different expression between HCM group and control group (FC > 2.0, *P*-value < 0.05 and FDR < 0.05) (Additional file 2). Among them, 819 mRNAs were down-regulated and 896 mRNAs were up-regulated. Their distinct expression patterns were presented by hierarchical clustering analysis (Fig. 2).

### Real-time PCR validation of some differentially expressed lncRNAs and mRNAs

**Fig. 3:**
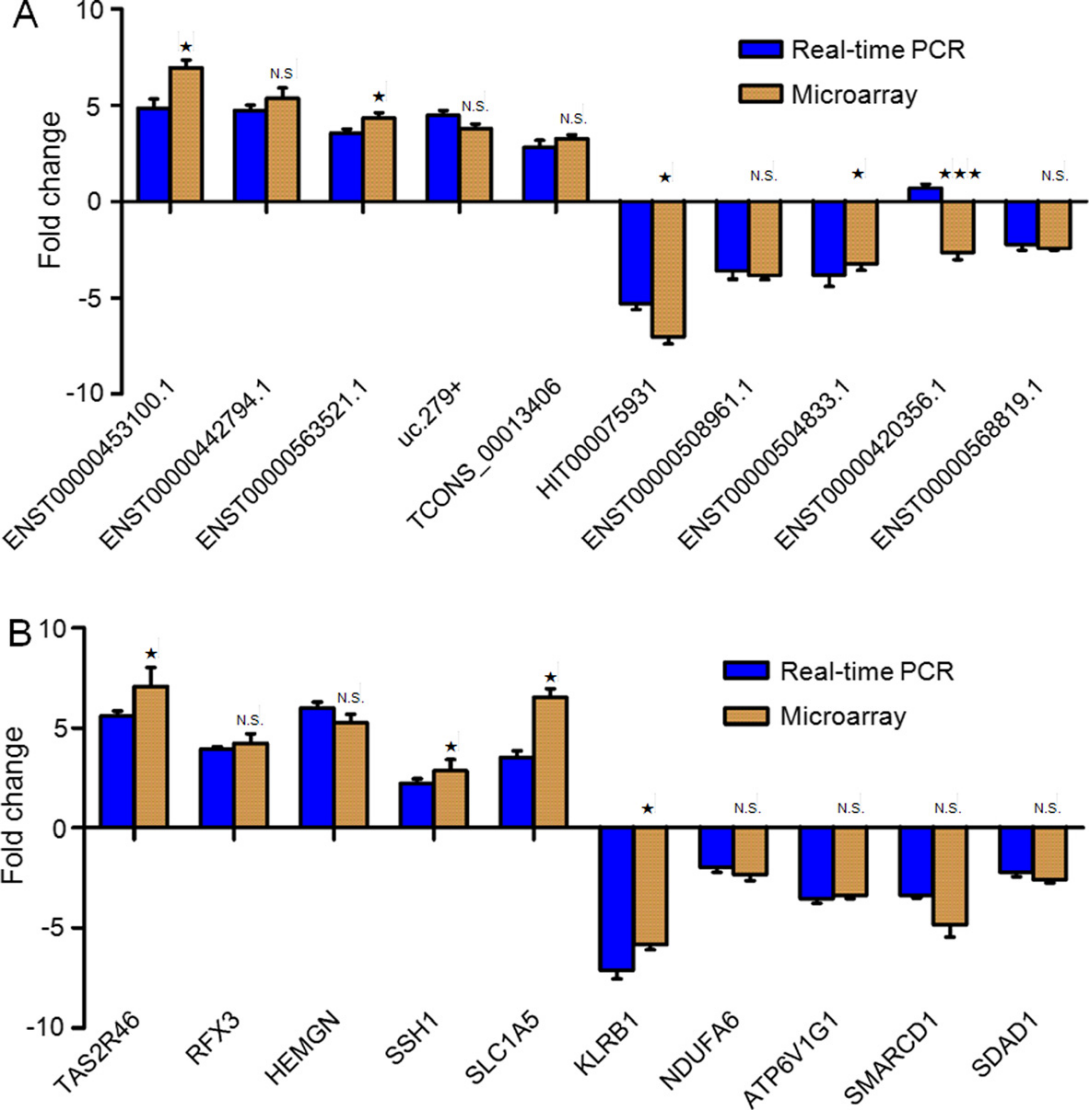
Real-time PCR validation of some differentially expressed lncRNAs and mRNA from microarray data. **a** 10 lncRNAs were randomly chosen for real-time PCR validation. **b** 10 mRNAs were randomly chosen for real-time PCR validation. Fold changes were calculated by the 2^-△△Ct^ method. Data shown are representative of 7 patients and 5 controls; error bars represent means ± SEM, ★*P* < 0.05, ★★*P* < 0.01, ★★★*P* < 0.001, N.S. not significant (student *t* test). Apart from ENST00000420356.1, the real-time PCR validation results of all lncRNAs and mRNAs were generally consistent with microarray data

We randomly selected 10 dysregulated lncRNAs, including 5 up-regulated (ENST00000453100.1, ENST00000442794.1, ENST00000563521.1, uc.279+ and TCONS_00013406) and 5 down-regulated (HIT000075931, ENST00000508961.1, ENST00000504833.1, ENST00000420356.1 and ENST00000568819.1), for verification in these myocardial tissues samples. A general consistency between the real-time PCR and microarray analysis results was confirmed in 9 selected lncRNAs in terms of regulation direction (up-regulation or down-regulation) and significance except ENST00000420356.1 (Fig. 3a).

Moreover, we randomly selected 10 dysregulated mRNAs, consisting of 5 up-regulated (TAS2R46, RFX3, HEMGN, SSH1 and SLC1A5) and 5 down-regulated (KLRB1, NDUFA6, ATP6V1G1, SMARCD1 and SDAD1). All the 10 mRNAs showed the same change patterns as shown in microarray analysis (Fig. 3b).

### GO and pathway analysis for differentially expressed mRNAs

**Fig. 4:**
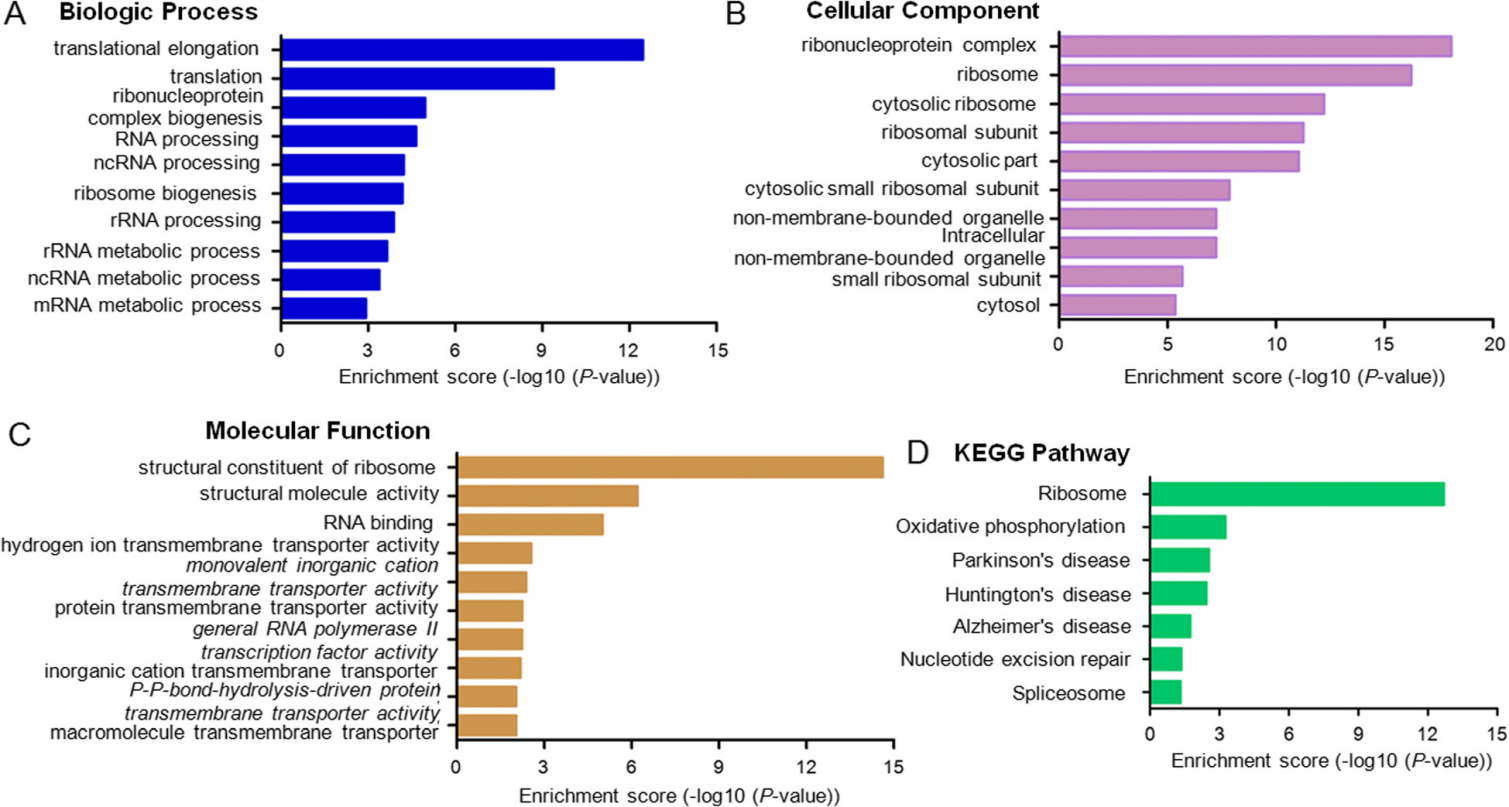
Enrichment analysis of pathways and GO terms for differentially expressed mRNAs. **a**-**c** GO analysis according to biological process, cellular component and molecular function, respectively. **d** Pathway analysis based on the KEGG database. *P*-values < 0.05 using the two-sided Fisher’s exact test was defined to be statistically significant

The GO categories for each gene were derived from the GO website (www.geneontology.org) and comprised of 3 structured networks: biological processes, cellular components and molecular function. Through GO analysis, we found that the differentially expressed mRNAs were principally enriched for translational elongation, translation, ribonuclearprotein complex biogenesis linked with biological processes (Fig. 4a), and ribonucleoprotein complex, ribosome, cytosolic ribosome involved in cellular components (Fig. 4b), as well as structural constituent of ribosome, structural molecule activity, RNA binding in molecular functions (Fig. 4c). Pathway analysis was carried out based on the KEGG database. The dysregulated mRNAs were associated with 12 biological pathways, including ribosome, oxidative phosphorylation, parkinson’s disease, huntington’s disease, alzheimer’s disease, as well as others (Fig. 4d).

**Fig. 5:**
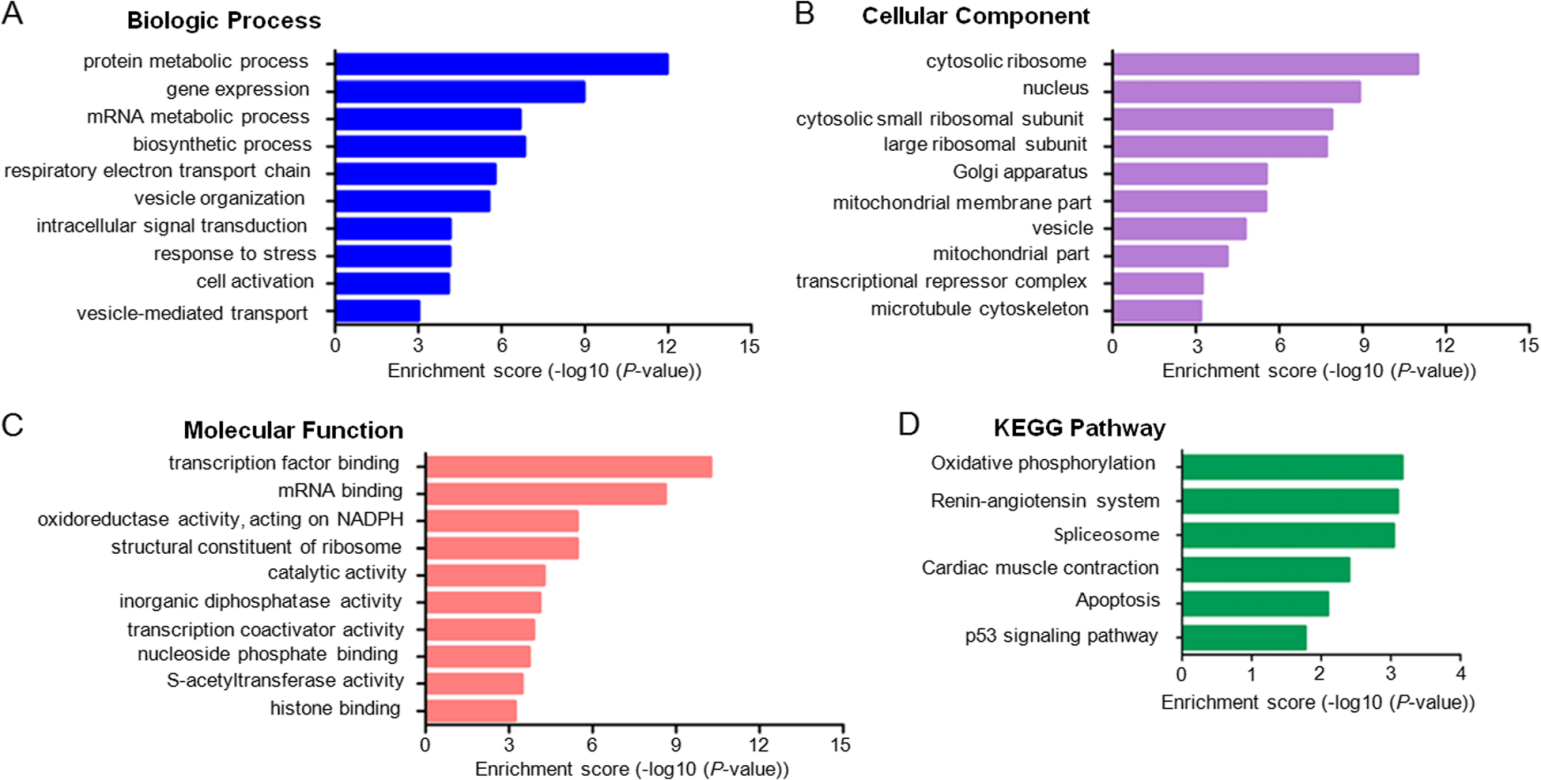
Enrichment analysis of pathways and GO terms for protein-coding neighbors of dysregulated lncRNAs. **a**-**c** GO analysis according to biological process, cellular component and molecular function, respectively. **d** Pathway analysis based on the KEGG database. *P*-values < 0.05 using the two-sided Fisher’s exact test was defined to be statistically significant

Several lines of evidence revealed that most of lncRNAs may act in cis and regulate gene expressions within their chromosomal neighboring regions [19]. We first identified the nearest protein-coding neighbor within 100 kb of dysregulated lncRNA and then applied GO and pathway analysis to determine the roles of these closest coding genes. The neighbor coding gene function of dysregulated lncRNAs mainly involved: (1) biological process (protein metabolic process, gene expression, mRNA metabolic process, biosynthetic process, etc.); (2) cellular component (cytosolic ribosome, nucleus, cytosolic small ribosomal subunit, large ribosomal subunit, etc.); (3) molecular function (transcription factor binding, mRNA binding, oxidoreductase activity, structural constituent of ribosome, etc.) (Fig. 5a–c). The neighbor gene function of dysregulated lncRNAs mainly involved the following pathways: (1) oxidative phosphorylation; (2) renin-angiotensin system; (3) spliceosome; (4) cardiac muscle contraction; (5) apoptosis; (6) p53 signaling pathway (Fig. 5d).

## Discussion

In the present study, we performed a comprehensive analysis of dysregulated lncRNAs by comparing the transcriptome profiles of hypertrophied myocardial tissues from HCM patients and normal myocardial tissues. A total of 8435 lncRNAs and 8404 mRNAs were detected. We identified 965 up-regulated and 461 down-regulated lncRNAs, and summarized their general characteristics and functional annotations. Thus, our study could provide a comprehensive understanding of lncRNAs in HCM patients and help to elucidate the molecular mechanisms of HCM.

To our knowledge, several proteomic and transcriptome profiling of HCM have been performed and a number of dysregulated genes have been identified before [20–23] (Additional file 3). For example, Lim et al. identified 36 dysregulated genes involved in cytoskeletal proteins, protein synthesis, redox system, ion channels and those with unknown function in HCM [20]. We compared the list of HCM-related genes between our study and previous studies and found that some genes appeared both in our study and previous studies, including *AFG3L2, AGPAT9, CDC14B, CMKLR1, COX4I1, GFM1, GLUD1, LDHB, LYRM4, MRPL22, NCAM1, SLC25A46, SS18L2, TACO1, TMEM135, TMEM41B and ZFAND1*.

However, there were also many genes which occurred only in previous studies but not in our study. We believed that discrepancy of expression profiling of mRNA and lncRNA between our study and previous studies was mainly derived from the following several reasons. First, as we know, the RNA and protein profiling fluctuated dramatically during different stages of diseases’ progress [24, 25]. In different progess stage of HCM, the RNA profiling might be variable and this would be the major reason. Second, time-dependent degradation of nucleic acids confers great difficulty to expression analysis when postmortem tissues were used. In our study, the normal myocardial tissues were excised from healthy formalin-fixed postmortem individuals 3–7 days after death, which might lead to significant RNA degradation [26, 27]. Inhomogenous RNA degradation would result in the change of relative expression of RNAs, Third, different detection platforms may have huge impact on the profiling result and this is why subsequent experimental verification is essential. Most of the previous studies used Affymetrix platform for profiling analysis, while, in our study, Arraystar Human LncRNA Microarray v2.0 was used. The molecular mechanisms responsible for HCM have been extensively explored. However the pathogenesis of this disease is still largely unknown. Most of the genes discovered in HCM were protein-coding while non-coding genes were scarcely reported. Technological improvements have contributed to reveal the importance of lncRNA underling various diseases [28]. Up till now, both transcriptome sequencing [29] and microarrays [30] have been applied to identify differential lncRNA expression. To the best of our knowledge, only one lncRNA, *Myheart*, was reported to be involved in cardiac hypertrophy in previous studies. *Myheart* could protect heart from pathological hypertrophy by its interaction with chromatin [31, 32]. Our study could greatly enrich the lncRNAs spectrums involved in the pathogenesis of HCM.

The Gene Ontology project provides a controlled vocabulary to describe gene attributes unbiasedly [18]. Interestingly, in our study, these dysregulated genes were mainly involved in translational regulation, including translational elongation, ribonucleoprotein complex, ribosome and structural constituent of ribosome. As we know, once the translational process was disrupted, the levels of numerous proteins would be influenced, which might have profound effects on normal physiological processes [33, 34]. However, no previous study of HCM was concentrated on translational regulation and we hold the opinion that it would be quite meaningful to investigate translational dysregulation in HCM. Pathway analysis identified 7 distinct pathways, including ribosome and oxidative phosphorylation. `It has been reported that CREB contributes to physiological hypertrophy by enhancing expression of genes in complex I of oxidative phosphorylation [35]. Many more studies are needed to fully understand the molecular mechanisms of HCM in the future.

## Conclusion

LncRNAs are involved in the pathogenesis of HCM through the modulation of multiple pathogenetic pathways. Our study could also provide clinic evidence for the diagnosis and prognostic management of HCM patients, as well as medical intervening for HCM.

### Data availability

The authors confirm that all data underlying the findings are fully available without restriction. All relevant data is in a public repository (GEO series accession number GSE68316 (http://www.ncbi.nlm.nih.gov/geo/query/acc.cgi?acc=GSE68316).

## Additional files

Additional file 1:
**Dysregulated lncRNAs between controls and HCM patients.**
Additional file 2:
**Dysregulated mRNAs between controls and HCM patients.**
Additional file 3:
**Brief summary of known mRNAs which have been found to be involved in pathogenesis of HCM.**
